# Broader roles of the ubiquitin-like protein Hub1 indicated by its yeast two-hybrid interactors

**DOI:** 10.17912/micropub.biology.000519

**Published:** 2022-01-25

**Authors:** Amjadudheen Varikkapulakkal, Anuraag Ghosh, Shravan Kumar Mishra

**Affiliations:** 1 Department of Biological Sciences, Indian Institute of Science Education and Research (IISER) Mohali, Sector 81, 140306 Punjab, India

## Abstract

The conserved ubiquitin-like protein Hub1/UBL5 functions in RNA splicing, DNA repair and mitochondrial unfolding responses. It binds proteins specific to these pathways and modifies their functional properties. However, the identities of other Hub1 substrates remain unknown. We have found unreported interactors of *Saccharomyces cerevisiae* Hub1 from a yeast two-hybrid (Y2H) screen. Proteins containing SIMs (small ubiquitin-like modifier SUMO-interaction motifs) and ferulic acid decarboxylase Fdc1 are identified as potential Hub1 interactors. Further experiments are required to establish these interactions and their physiological relevance, nevertheless, data presented here point towards larger and intriguing roles of Hub1.

**Figure 1. Potential interactors of Hub1 identified from the Y2H screen:**
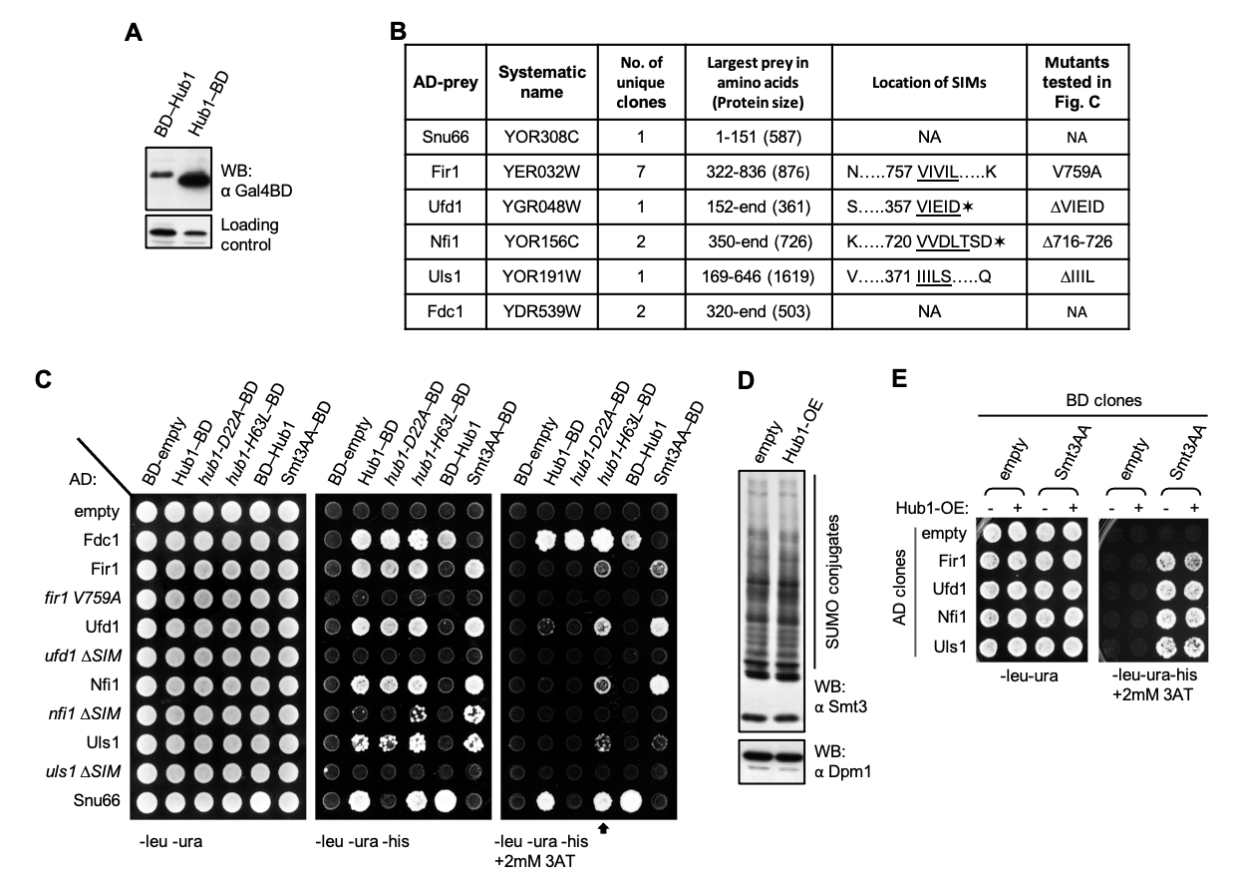
**(A)** Expression of Hub1 fusion clones with N- or C- terminal Gal4-BD. Proteins from total yeast lysate were detected by a monoclonal antibody specific to Gal4-BD. BD at Hub1’s C-terminus is detectable in larger amounts possibly because of a chaperone-like activity of Hub1 on C-terminal extensions (chaperoning activities are known for ubiquitin and SUMO). Loading control represents an antibody cross-reactive signal of unknown identity detected in the lysates. **(B)** Clones obtained from Y2H screen. Hub1-Snu66 interaction was reported previously. Preys encoding Fir1, Ufd1, Nfi1, and Uls1, known to non-covalently bind yeast SUMO (Smt3) through their SIMs (underlined), were found as Hub1 interactors. SIM mutants used in Fig. C are indicated. In addition, two independent clones of Fdc1 were also obtained as Hub1 interactors (NA, not applicable, proteins lack SIM). Asterisks indicate stop codons. **(C)** Targeted Y2H assays monitoring interactions between indicated baits and the largest prey clones obtained in the screen. Each spot represents one of the 66 combinations of baits and preys co-transformed in the yeast. Full length (FL) Snu66 was used as prey control, which as expected interacted with Hub1 but not the D22A mutant. Yeast SUMO with its di-glycine motif changed to di-alanine (Smt3AA) was used as a control for monitoring SIM binding. Point mutant or deletions of SIMs in Fir1, Ufd1, Nfi1, Uls1 defective in Smt3AA binding did also not bind Hub1. SIM binding to *hub1-D22A* mutant was unaltered, while the *H63L* mutant showed stronger interactions (indicated by an open arrow). The N-terminal BD-Hub1 fusion did not bind SIM-containing clones either due to lower protein expression (Fig. A) or because these interactions require a free N-terminal surface of Hub1. By contrast, binding to Snu66 and Fdc1 was unaffected by BD’s location. **(D)** Covalent SUMO conjugates (detected by anti-Smt3 antibody) were unaltered in yeast overexpressing (OE) Hub1. **(E)** Y2H assays between indicated baits and preys upon Hub1 overexpression. SUMO-SIM interactions were not weakened upon Hub1 overexpression.

## Description

Hub1/UBL5 has been reported to function in pre-mRNA splicing (Chanarat and Mishra, 2018; Wilkinson *et al.*, 2004), DNA repair (Oka *et al.*, 2015) and mitochondrial unfolding responses (Benedetti *et al.*, 2006). It associates with proteins only non-covalently thereby modifying their functional properties (Chanarat, 2021). Through its Asp-22 surface Hub1 binds HIND segments on the splicing factor Snu66/SART1 and through its His-63 surface Hub1 binds the RNA helicase Prp5 required for spliceosome formation (Karaduman *et al.*, 2017; Mishra *et al.*, 2011). An earlier study of Hub1 adducts in *S. cerevisiae* has indicated that this UBL may also have a larger number of substrates (Lüders *et al.*, 2003). However, identifying Hub1 substrates has been challenging, possibly due to its transient, weak or substoichiometric associations. We have performed a Y2H screen using *S. cerevisiae* Hub1 as bait fused to the DNA binding domain (BD) of the Gal4 transcription factor at the C-terminus. This fusion raises the possibility of identifying substrates binding through the N-terminal surface. Also, this fusion protein accumulated to higher levels in yeast (Fig. A).

In Y2H screens, Gal4BD fused to the protein of interest is used as bait for identifying its binding partners from a library of preys (DNA clones fused to the transcription activation domain (AD) of the same transcription factor). Once the bait binds a prey, functional Gal4 transcription factor gets reconstituted, whose activity is measured by various *GAL* promoters driven reporters, for example, *HIS3* (conferring histidine auxotrophy), *ADE2* (conferring adenine auxotrophy) (James *et al.*, 1996). Screens with partial and random clones become more useful when full-length baits and preys perform poorly due to improper expression, folding, or non-nuclear localisation of fusion proteins. Preys used in the screen were Gal4AD coding sequence fused to a random *S. cerevisiae* genomic DNA library in all three reading frames. ~5×10^5^ bait-prey co-transformed yeast were screened for each reading frame. Besides the previously reported splicing factor Snu66, clones encoding Fir1, Nfi1, Uls1, and Ufd1 with their SIMs, and Fdc1 were identified as Hub1 interactors (Fig. B).

SIMs are found in proteins with diverse roles. Through a short stretch of hydrophobic amino acids with an acidic region, often unstructured SIMs bind SUMO non-covalently. Upon binding, SIMs adopt partial beta-strand conformations (Lascorz *et al.*, 2021). Among the clones containing SIMs identified here, Fir1 is involved in 3’ mRNA processing, Nfi1/Siz2 is a SUMO E3 ligase, Uls1/Ris1 is a SUMO-targeted ubiquitin ligase, and Ufd1 is a polyubiquitin-binding substrate recruiting cofactor of Cdc48. SIMs in Fir1, Nfi1, Uls1, and Ufd1 clones (Hannich *et al.*, 2005) were verified by their positive interactions with yeast SUMO, whereas their SIM mutants were defective in SUMO binding. Smt3 in general interacted more strongly with SIM-containing clones than Hub1. Hub1-specific substrates Snu66 and Fdc1 lacked SIM and did not bind Smt3 (Fig. C). *hub1-D22A* mutant defective in Snu66 binding (Ammon *et al.*, 2014; Mishra *et al.*, 2011) interacted normally with SIM-containing clones, suggesting SIM associates to another surface of Hub1. On the contrary, the *hub1-H63L* mutant defective in binding to the splicing RNA helicase Prp5 (Karaduman *et al.*, 2017) interacted strongly with SIMs (Fig. C). The Leu63 variant’s improved interactions suggest a hydrophobic mode of association with SIM and potential overlap with the Prp5-binding site. SIM point mutation, or deletions of the hydrophobic regions abrogating SUMO binding, did also not bind Hub1 (Fig. C), thereby indicating similar hydrophobic interactions between Hub1 and SIMs. The data also indicate potential promiscuity in SIMs. The hydrophobic associations of Hub1 possibly represent some of its adducts reported previously (Lüders *et al.*, 2003).

Notwithstanding the above evidence, binding sites for Smt3/SUMO and Hub1 on these SIMs need to be defined better by making point mutants of SIMs and comparing their interactions with the two UBLs. Similarly, SIM binding site on Hub1 needs to be defined better. Furthermore, SIMs even though are found in unstructured segments of proteins (Lascorz *et al.*, 2021), where point mutations and deletions are less likely to alter overall structures, protein folding and stability issues for the mutants need to be ruled out. The overlapping binding sites of Hub1 and SUMO suggested potential competition in the non-covalent associations of the two UBLs. However, owing to stronger SUMO-SIM associations, Hub1 overexpression neither altered covalent SUMO conjugates (Fig. D) nor the non-covalent SUMO-SIM binding (Fig. E). However, these assays are not highly quantitative or physiological for ruling out potential crosstalk between Hub1 and SUMO. Further biochemical assays are needed to confirm Hub1-SIM associations and follow up studies would reveal the function and mechanism of Hub1 with these molecules.

Fdc1-Hub1 interaction was not affected in the *hub1-D22A* mutant. Fdc1 lacks SIM and did not bind SUMO. Thus, Hub1 interaction with Fdc1 is distinct from its interaction with Snu66 or SIMs, for which another yet to be identified surface of Hub1 might be used (Fig. C). Diverse applications of ferulic acid (Kumar and Pruthi, 2014) make the Hub1-Fdc1 association an interesting research subject. Taken together, our data indicate that Hub1 employs additional surfaces for protein-protein interactions. Through hydrophobic associations, Hub1 might play broader roles that include possible crosstalk with other UBLs and potential decarboxylation of aromatic carboxylic acids.

## Methods

Strains and plasmids used for yeast two-hybrid screens are as described previously (James *et al.*, 1996). The hub1 deletion strain was described earlier (Mishra *et al.*, 2011). Standard yeast protocols were followed for yeast transformation, growth assays and protein analysis (Knop *et al.*, 1999).

**Western blots:** A monoclonal antibody specific for Gal4-BD (Clonetech) was used for monitoring the expression of BD fusions in total yeast lysates. Smt3/SUMO conjugates were monitored in total yeast lysates using an anti-Smt3 polyclonal antibody. Cells were treated with 10mM NEM for 1 hour prior to harvesting. Preparation of sample for western blot analysis was described before (Knop *et al.*, 1999).

**Plasmids construction:** Plasmids were constructed through restriction enzyme cloning to ligate desired inserts into appropriate vector backbones following their amplification from *S. cerevisiae* genomic DNA using specific primer sets. The pGBDU-C1 plasmid was used to prepare bait clones with N-terminal BD. C-terminal BD clones were prepared in a uracil positive YEplac195 vector by inserting ADH promoter (between EcoRI and BamHI restriction sites), Hub1 or Smt3AA coding sequences (between BamHI and PstI restriction sites), BD coding sequence (at PstI restriction site), and ADH terminator (between PstI and SphI restriction sites), in that order. Random genomic DNA libraries were prepared in all three coding frames in pGAD-C1, C2 and C3 plasmids by inserting yeast genomic DNA fragments digested with ClaI restriction enzyme (James *et al.*, 1996). All prey clones and their mutants were made in the pGAD-C1 plasmid. The point mutants were made by quick-change site-directed mutagenesis and SIM deletions through splicing by overlap extension (SOE) PCR or by inserting a premature stop codon in the C-terminally located SIM of Ufd1. Hub1 was overexpressed either by using a centromeric vector with TEF2 promoter or an episomal vector with ADH promoter.

**Yeast two-hybrid screen and assays:** Y2H screen was performed essentially following published protocols (Gietz *et al.*, 1997; James *et al.*, 1996). In brief, the *S. cerevisiae* strain PJ69-7A was transformed with Hub1-BD bait and transformants were selected on synthetic complete media lacking uracil. The bait transformed yeast was cultured, made competent and transformed with the leucine positive Gal4AD genomic DNA libraries at 60X scale in all three reading frames. An aliquot was plated on media lacking uracil and leucine to estimate the total number of transformants screened. The remaining transformation mixture (~5×10^5^ transformants in each reading frame) were plated on media lacking uracil, leucine and histidine (–ura –leu –his) to select putative positive interactors. After 5 days of incubation at 30°C, putative positive colonies were replica plated on different reporter plates lacking histidine with/out 3AT and lacking adenine. For autoactivation test of prey fusions, first positive colonies were streaked on –leu + 5-FOA plates to shuffle out the bait marked with uracil, then autoactivation test of prey-containing colonies was done by streaking on –leu –his plates. AD plasmids from non-autoactivating colonies were isolated by yeast shuttle prep. Prey clones showing positive interaction after retransformation with the bait were sequenced. For targeted Y2H assays, the PJ69-7A strain was co-transformed with different combinations of AD and BD clones. The transformants were selected by plating on appropriate synthetic dropout plates and by growing them at 30°C for 2-3 days. Cells from each co-transformants were suspended in 500µl sterile waterand their OD_600_ was measured. Appropriate volumes of sterile water and the cell suspensions were mixed in a 96-well microtiter plate to get a final cell density of 1.0 OD_600_ / ml. 0.005 OD_600_ cells were spotted on various selection plates. The spotted cells were allowed to grow at 30°C for 3 days.
